# Constituents’ Inferences of Local Governments’ Goals and the Relationship Between Political Party and Belief in COVID-19 Misinformation: Cross-sectional Survey of Twitter Followers of State Public Health Departments

**DOI:** 10.2196/29246

**Published:** 2022-02-10

**Authors:** Hannah Stevens, Nicholas A Palomares

**Affiliations:** 1 Department of Communication College of Letters and Science University of California, Davis Davis, CA United States; 2 Department of Communication Studies Moody College of Communication The University of Texas at Austin Austin, TX United States

**Keywords:** COVID-19, outbreak, mass communication, Twitter, goal inferences, political agendas, misinformation, infodemic, partisanship, health information

## Abstract

**Background:**

Amid the COVID-19 pandemic, social media have influenced the circulation of health information. Public health agencies often use Twitter to disseminate and amplify the propagation of such information. Still, exposure to local government–endorsed COVID-19 public health information does not make one immune to believing misinformation. Moreover, not all health information on Twitter is accurate, and some users may believe misinformation and disinformation just as much as those who endorse more accurate information. This situation is complicated, given that elected officials may pursue a political agenda of re-election by downplaying the need for COVID-19 restrictions. The politically polarized nature of information and misinformation on social media in the United States has fueled a COVID-19 infodemic. Because pre-existing political beliefs can both facilitate and hinder persuasion, Twitter users’ belief in COVID-19 misinformation is likely a function of their goal inferences about their local government agencies’ motives for addressing the COVID-19 pandemic.

**Objective:**

We shed light on the cognitive processes of goal understanding that underlie the relationship between partisanship and belief in health misinformation. We investigate how the valence of Twitter users’ goal inferences of local governments’ COVID-19 efforts predicts their belief in COVID-19 misinformation as a function of their political party affiliation.

**Methods:**

We conducted a web-based cross-sectional survey of US Twitter users who followed their state’s official Department of Public Health Twitter account (n=258) between August 10 and December 23, 2020. Inferences about local governments’ goals, demographics, and belief in COVID-19 misinformation were measured. State political affiliation was controlled.

**Results:**

Participants from all 50 states were included in the sample. An interaction emerged between political party affiliation and goal inference valence for belief in COVID-19 misinformation (∆*R*^2^=0.04; *F*_8,249_=4.78; *P*<.001); positive goal inference valence predicted increased belief in COVID-19 misinformation among Republicans (β=.47; *t*_249_=2.59; *P*=.01) but not among Democrats (β=.07; *t*_249_=0.84; *P*=.40).

**Conclusions:**

Our results reveal that favorable inferences about local governments’ COVID-19 efforts can accelerate belief in misinformation among Republican-identifying constituents. In other words, accurate COVID-19 transmission knowledge is a function of constituents' sentiment toward politicians rather than science, which has significant implications on public health efforts for minimizing the spread of the disease, as convincing misinformed constituents to practice safety measures might be a political issue just as much as it is a health one. Our work suggests that goal understanding processes matter for misinformation about COVID-19 among Republicans. Those responsible for future COVID-19 public health messaging aimed at increasing belief in valid information about COVID-19 should recognize the need to test persuasive appeals that address partisans’ pre-existing political views in order to prevent individuals’ goal inferences from interfering with public health messaging.

## Introduction

### Background

Amid the widespread global COVID-19 pandemic, social media have exacerbated the spread of health misinformation and disinformation [1]; belief in false health information is, at times, just as common as the endorsement of accurate information [2]. The politicized and polarized state of information surrounding COVID-19 in the United States has fueled a concomitant infodemic on social media, where “facts” are subjective depending on one’s political agenda [3-7].

Public health agencies often use Twitter as a tool to disseminate and amplify the propagation of COVID-19 information [8,9], but exposure to local government–endorsed public health information via Twitter does not make one immune to believing COVID-19 misinformation. Whereas public health agencies, via their Twitter accounts, can share valid information and details of their concerted efforts to protect constituents, politicians are equally likely at times to distribute misinformation via tweets to pursue political agendas that could harm their constituents [10,11]. In fact, incongruencies in tweets exist in COVID-19 messaging across unique state public health agencies’ and individual stakeholders’ Twitter accounts [8].

Whereas conservative rhetoric connected to Republican politicians is associated with more misinformation, democratic rhetoric is more consistent with guidelines from public health officials [2,12,13]. As a result, US partisan affiliation is a stronger predictor of COVID-19 beliefs than local infection rates or demographics (eg, health status and age) [14]. Yet, the relationship between Republican partisanship and COVID-19 misinformation is nuanced when considering the potential goal understanding processes at work. Despite the high levels of COVID-19 misinformation, red state partisans are largely dissatisfied with their state government’s management of the pandemic; this low approval of their state politicians’ efforts is even more depressed for politicians who have been resistant to implementing business closures as a safety measure [14]. Thus, many Republicans with red viewpoints are unhappy with what their state government has done to effectively manage the pandemic. However, the goal understanding processes that facilitate belief in COVID-19 misinformation are unclear.

### Theoretical Framework

Pre-existing political beliefs can influence the endorsement of misinformation [3,15-17]. In the context of the COVID-19 pandemic, politicians from the Republican Party and right-leaning media figures downplayed the threat of COVID-19 in comparison to Democratic politicians and left-leaning media figures while focusing on the economic damages resulting from widespread business closures and the threat to individuals’ personal liberties [18,19]. As a result, media and political figures’ attitudes toward the COVID-19 pandemic cascaded to Republican supporters, affecting individuals’ compliance with public health guidelines, including mask wearing and social distancing [3,19-21]. Given that extant research suggests that Republicans are exposed to more persuasive messages containing misinformation from their party leaders compared to Democrats [3,17-21], we posit the following hypothesis: Republicans endorse greater levels of COVID-19 misinformation than Democrats (hypothesis 1 [H1])*.*

When Republicans experience discontent with their local government’s public health efforts however, their endorsement of COVID-19 misinformation is reduced. If Republicans think that their local government is not doing a good job and perhaps think that the government is serving a less prosocial agenda, then they will believe less misinformation about COVID-19. According to goal understanding theory, the goal inferences that people make about others have spillover effects or consequences beyond merely endorsing a goal inference [22]. We theorize that the association between increased discontent and the decreased endorsement of misinformation occurs because Republicans are likely relatively more critical of their government and its efforts when their goal inferences are negatively valenced. This spillover effect for Republicans’ inferences of their local government’s goals results in the more systematic processing of relevant persuasive messages promoting misinformation about COVID-19 and thus reduces their endorsement of such beliefs spread by party leaders. On the other hand, when Republicans think that their local government is doing well and they have positive sentiments toward their government’s agenda, they tend to endorse more COVID-19 misinformation, given the conservative ideologies related to COVID-19. However, we do not expect to find this same spillover effect for Democrats’ goal understanding processes because of the reduced likelihood that they endorse misinformation on COVID-19, given the focus on science-based practices associated with liberal political beliefs regarding the pandemic. In other words, political affiliation likely interacts with goal inference valence in ways that matter for belief in misinformation, as we predict herein: Republican Twitter users’ positive goal inference valence for their local government’s COVID-19 efforts predicts heightened belief in COVID-19 misinformation, whereas this outcome is not the case for Democrats (hypothesis 2 [H2]).

We are uncertain about the relationship between goal inference valence for government COVID-19 efforts and belief in misinformation about SARS-CoV-2 for independents or those without a political affiliation. Indeed, independent voters generally lean toward 1 of the 2 major partisan ideologies; 48% of independents leaned Democrat and 54% leaned Republican as of 2019 [23]. Americans who do not lean toward a particular party are less politically informed [23]. Thus, individuals who do not identify with a party may not be influenced by mediated messages from politicians to the same extent as partisans. Similarly, independents have more negative sentiments toward political parties and politicians [23]. As such, they may be less susceptible to believing politicized misinformation. Yet, we refrain from generating predictions and propose the following research question: what is the relationship between the goal inference valence for local governments’ COVID-19 efforts among independent Twitter users and those with other or no party affiliations and their belief in COVID-19 misinformation?

## Methods

### Study Design

We conducted a web-based cross-sectional survey of US Twitter users (n=258) who followed their state’s official Department of Public Health Twitter account between August 10 and December 23, 2020. The valence of inferences about local governments’ goals, demographics, and belief in COVID-19 misinformation were measured. We controlled for state political affiliation based on the 2020 presidential election outcome. We conducted a linear regression analysis to assess whether political party and the valence of inferences about state governments’ goals significantly predicted belief in COVID-19 misinformation while controlling for state party affiliation. The institutional review board of University of California, Davis (protocol number: 1502267-5), approved all study materials and procedures prior to data collection.

### Recruitment

We took a random sample of Twitter users who follow their state’s official Department of Public Health Twitter account (eg, California Department of Public Health, Oregon Department of Public Health, etc). Each state’s Department of Public Health has an official Twitter account. These Twitter accounts received an influx of social media engagement in 2020, which was likely due to concern regarding COVID-19. Consequently, it is likely that each state’s followers were impacted by COVID-19 social distancing measures and that users are following their state’s Department of Public Health because they are interested in information about COVID-19 for the state in which they reside.

We randomly selected the final sample of 200 participants from each of the 50 states (200 × 50 = 10,000) from a shuffled list of all followers from each state’s Department of Public Health. Then, we distributed the hyperlink to the survey to each follower in our sampling frame (n=10,000) through Twitter and asked our sample of participants to respond. Of the 10,000 members of our sample, 532 (5.3%) responded to our direct message. This nonresponse level was expected; whereas research shows that traditional telephone response rates are low (<10%), response rates in web-based communities are reported to be even lower (<6%) [24,25].

### Survey Development

This cross-sectional survey consisted of demographic questions, measures of COVID-19 knowledge, questions related to political party affiliation, and an open-ended question asking participants about their local government’s crisis response goals. COVID-19 misinformation items were selected by comparing the Centers for Disease Control and Prevention guidelines for slowing the spread of COVID-19 with common, prevalent COVID-19 myths [26-28]. Multimedia Appendix 1 contains details for the open-ended goal inference measure and the misinformation items. Qualtrics programming software (Qualtrics International Inc) was used to host the survey. Prior to data collection, an expert in survey design reviewed all measures for their effectiveness, and we made adjustments based on the expert’s feedback.

### Procedure

We sent (ie, via direct message) our sampling frame an invitation to participate in the survey. Participants who clicked the survey link were directed to an electronic consent form. Of the 10,000 followers messaged, 532 participants consented to participate.

Participants were asked a series of questions about their inferences of their local government’s goals, demographics, political party identification (Democrat, Republican, independent, or other), and COVID-19 misinformation (Multimedia Appendix 1). State political affiliation was controlled. Of the 532 participants who consented, 274 were excluded from the final analysis because they did not complete more than 1 item; 258 participants were retained.

### Statistical Analysis

#### COVID-19 Misinformation Computation

We computed the endorsement of COVID-19 misinformation by calculating the sum of the number of myths (5 myths in total) that each participant endorsed and the sum of the number of truths (5 truths in total) about SARS-CoV-2 that they did not endorse. Each myth and truth was effectively coded as “1” for having a false belief or as “0” for having an accurate belief. In other words, if people believed all 5 falsehoods about COVID-19 and rejected all 5 truths, then their score would be 10, which is the theoretical maximum, whereas those rejecting all falsehoods and accepting all truths would yield a score of 0—the theoretical minimum. On average, participants believed 1.27 (SD 1.21, SE 0.08; minimum=0; maximum=5.00; skewness=1.01; kurtosis=0.64) myths.

#### Valence of Inferences About Local Governments’ Goals

Participants' open-ended textual inferences of their government’s goals were processed through the Linguistic Inquiry and Word Count (LIWC) computerized text analysis tool [29] to quantify the emotional valence of each participant’s open-ended goal inference [22]. LIWC uses raw word counts to assign scores to texts in psychology-relevant categories, including scores for the emotional tone (ie, valence) of a text, and it has been used in recent medical internet research to measure emotion in textual responses, including sentiment toward the COVID-19 pandemic [30-35]. LIWC assigns each text an emotional valence score based on the percentage of words used in the text by comparing the text to a dictionary of words in relevant categories. LIWC has been used in hundreds of studies and has been extensively validated (ie, via a word selection stage, an assessment of the base rate of the frequency of words, and a phase in which human judges cross-validated the prior stages). Further, the program’s capabilities have undergone over 10 years of refinement [36]. LIWC emotional tone scores range from 1 to 100; a score of 100 indicates maximally positive emotional valence, and a score below 50 indicates more negative emotional valence. Participants’ average inference valence was negative, as their average tone score was 38.86 (SD 35.20, SE 2.19; minimum=1.00; maximum=99.00; skewness=0.86; kurtosis=−0.80).

#### State Partisan Affiliation

Participants from all 50 states were retained and included in our study. Between 1 to 15 participants came from each of the 50 states; Louisiana and Massachusetts had the most participants, with 13 (13/258, 5%) and 15 (15/258, 5.8%) participants, respectively. Each participant's self-reported state of residence was aggregated with the state’s partisan leaning during the 2016 presidential election [37]. Slightly over half of participants lived in red states (143/258, 55%).

## Results

The majority of participants were female (157/258, 60.9%), were White (212/258, 82.2%), and identified as a Democrat (129/258, 50%) or a Republican (66/258, 25.6%). The most frequently observed education level was a bachelor's degree from a college (81/258, 31.4%). The average participant age was 44.17 (SD 12.21, SE 0.76; minimum=19.00; maximum=75.00; skewness=0.11; kurtosis=−0.66) years.

The linear regression results for H1 revealed that while controlling for state political leaning, party affiliation was not a significant predictor of belief in COVID-19 misinformation (*P*=.66). Whereas the average score for belief in misinformation for Democrats was 1.11 (SD 1.17, SE 0.10; minimum=0; maximum=5.00; skewness=1.15; kurtosis=1.03), this value was twice as high for Republicans (mean 2.15, SD 1.37, SE 0.24; minimum=0; maximum=5.00; skewness=0.46; kurtosis=−0.55) and was in the expected direction. For independents, the average score for belief in misinformation was 1.21 (SD 1.12, SE 0.14; minimum=0; maximum=5.00; skewness=1.11; kurtosis=1.23); the average score for belief in misinformation for participants who reported another or no party affiliation was 1.13 (SD 1.07, SE 0.20; minimum=0; maximum=4.00; skewness=0.75; kurtosis=0.01). Overall, these results are inconsistent with H1.

When testing H2, the results revealed an interaction between political party affiliation and goal inference valence (∆*R*^2^=0.04; *F*_8,249_=4.78; *P*<.001). More positively valenced inferences of the government’s COVID-19 goals strengthened the relationship between party affiliation and belief in COVID-19 misinformation among Republicans when compared to that among Democrats (B=0.01; *t*_249_=2.03; *P*=.04), as predicted. Positive goal inference valence predicted increased belief in COVID-19 misinformation for Republicans (β=.47; *t*_249_=2.59; *P*=.01) but not for Democrats (β=.07; *t*_249_=0.84; *P*=.40).

**Figure 1:**
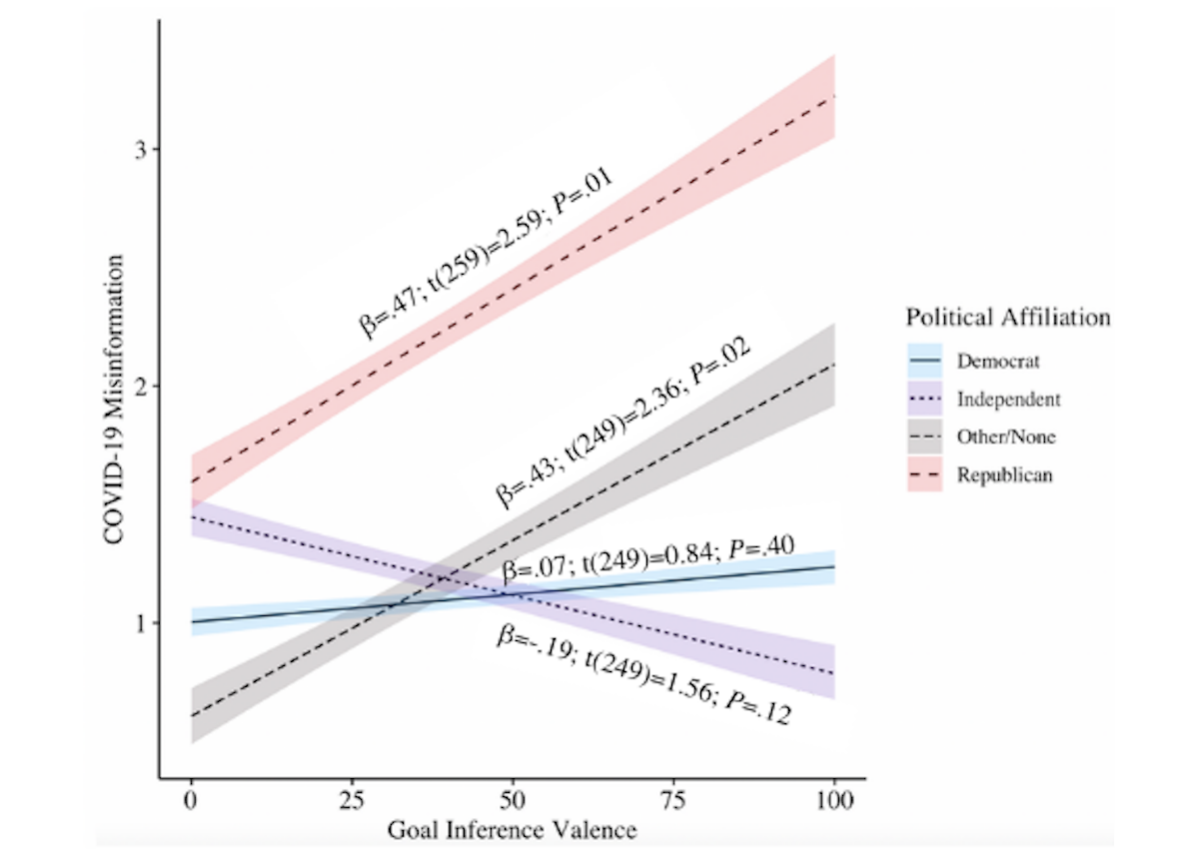
Interaction plot.

With regard to the research question, the relationship between goal inference valence and belief in misinformation is not significant for independents (β=−.19; *t*_249_=1.56; *P*=.12) and is significant in the positive direction for those with no party affiliation or another affiliation (β=.43; *t*_249_=2.36; *P*=.02). Table 1 shows the regression table, and Figure 1 shows a representation of the interaction.

**Table 1:** Regression results for political party interacting with goal inference valence to predict belief in COVID-19 misinformation^a,b^.

Variable	B (SE; 90% CI)	β	*t* (*df*)	*P* value
Intercept	0.98 (0.17; 0.70 to 1.25)	0	5.78 (249)	<.001
Blue state	0.06 (0.15; −0.18 to 0.31)	.03	0.44 (249)	.66
Inference valence	0 (0; 0 to 0.01)	.07	0.84 (249)	.40
Independent	0.44 (0.26; 0.02 to 0.87)	.16	1.72 (249)	.09
Other or no party affiliation	−0.40 (0.34; −0.97 to 0.17)	−.11	−1.15 (249)	.25
Republican	0.59 (0.33; 0.04 to 1.14)	.16	1.77 (249)	.08
Inference valence (independent)	−0.01 (0.01; −0.02 to 0)	−.17	−1.77 (249)	.08
Inference valence (other or no party affiliation)	0.01 (0.01; 0 to 0.02)	.17	1.82 (249)	.07
Inference valence (Republican)	0.01 (0.01; 0 to 0.03)	.19	2.03 (249)	.04

^a^*F*_8,249_=4.78; *P*<.001; *R*^2^=0.13.

^b^Unstandardized Regression Equation: COVID-19 misinformation = 0.98 + 0.06*blue state + 0*inference valence + 0.44*independent − 0.40*no or other party affiliation + 0.59*Republican − 0.01*inference valence (independent) + 0.01*inference valence (no or other party affiliation) + 0.01*inference valence (Republican).

## Discussion

This project examines the cognitive processes underlying the relationship between partisanship and health misinformation. We investigate how positive sentiment toward local governments’ COVID-19 efforts can enable or impede belief in COVID-19 misinformation.

### Principal Results

Our results reveal that even though the overall endorsement of misinformation regarding COVID-19 does not vary across political party affiliations, when considering the valence of goal inferences among Republicans versus those among Democrats, a more complex pattern of results emerges. Republicans’ positive inferences about their local government’s COVID-19 efforts can accelerate belief in misinformation, given conservatism’s reliance on politics rather than science in their pandemic information dissemination efforts [4]. In other words, if Republicans believe that their local government has positive intentions, they may be more vulnerable to believing politically fueled COVID-19 misinformation than Democrats. As a result, accurate COVID-19 transmission knowledge has been driven by politicians’ political agendas and state partisan orientations rather than science. This is not the case for Democrats because of the science-based information campaigns of liberal political agendas [3]. Curiously, individuals without a mainstream political affiliation who had positive sentiment about their local government’s goals to address COVID-19 tended to endorse more misinformation, which is similar to Republicans. We speculate that this outcome was due to their lack of information. Indeed, US citizens who do not lean toward a particular party are relatively less politically informed [23]. At the same time, we recognize the exploratory nature of our work and understand that confirmatory work in the future is needed, especially when considering that party affiliation is not always associated with conservative views. Indeed, having no party affiliation yielded results consistent with those for Republicans; however, we caution that additional research is needed, as having no affiliation does not mean that one is apolitical.

### Limitations

Our study is subject to a few limitations. As with all cross-sectional studies, we do not have evidence for the direction of causality, even if theory suggests that there is a causal relationship between goal inference valence and the endorsement of misinformation about COVID-19. We also recognize that the goal understanding mechanisms underlying misinformation are likely more complicated, as they involve other constructs of theoretical significance such as rationality, which is an important factor in risk communication [38].

Previous work has also found that the LIWC computerized coding methodology may overidentify emotional expression [39]. Thus, LIWC may have captured extraneous sentiments when quantifying participants’ open-ended goal inferences.

Another limitation is participant self-selection, which suggests that participants who volunteered for this study were somehow motivated to share their thoughts about the topic. This makes them fundamentally different from those who opted to not participate. Although more Democrats (129/258, 50%) than Republicans (66/258, 25.6%) completed the survey, participants from red states tended to have a higher response rate (143/258, 55.4%) than those from blue states (115/258, 44.6%). This may be because liberals, who are living with stricter COVID-19 public health guidelines than conservatives who are living in red states with more relaxed guidelines, may be especially concerned about COVID-19. Consequently, non-Republicans living in red states may have been more incentivized to participate in this study and overrepresented [3,17]. These concerns are connected to our small sample size and use of Twitter as a recruitment means. Thus, we cannot generalize beyond our sample, especially if one considers participant self-selection to be a potential bias for our sample and findings. Future work is required to gain more confidence in our findings. At the same time, we purposefully recruited participants who follow their local health department’s Twitter account because we felt that these people would more likely be affected by partisan agendas than the general population; regardless, our findings should be interpreted with sampling limits in mind.

Although it was not practical to identify all potential confounders, we expect that inference valence varied among participants according to their personality, general trust for governments and their agencies, mental health risk factors, and exposure to COVID-19 information. To reduce bias through methodological triangulation, future work should complement our survey design with experimental data on exposing people to different government campaign messages and assessments of their goal inferences and COVID-19 beliefs and intentions. This may help extrapolate the specific sources of negative inference valence for governments’ goals regarding COVID-19 (eg, negatively valenced inferences resulting from mask and vaccine mandates vs less autonomy-restrictive messaging) and the role that goal understanding plays between governments and their constituents.

### Theoretical Implications

Despite these limitations, we find merit in our findings and think that they suggest several meaningful theoretical implications that are consistent with past work [1,7]. Goal understanding theory [22] was supported in the novel context of the government’s goal to address the COVID-19 public health crisis. Goal inferences are consequential for what people believe to be true about a global pandemic and how they might protect themselves, similar to how trust in science and politics can influence the measures that people take to protect themselves from SARS-CoV-2 infection [6]. Previously, goal inference mechanisms had only been demonstrated in personal relationships, such as those among friends or classmates, that have been dyadic [22,40]. Moreover, the spillover effects have been limited to more interpersonal processes without public health implications. Our research extends goal understanding spillover effects to the novel, hitherto unexplored context of the politicized endorsement of public health misinformation. Mechanisms that occur at the dyadic level of communication in close relationships likewise manifest in contexts where the agent and its goals function at a more macrosociological level of communication. Future research can expand on these theoretical implications by assessing how people understand the goals of specific politicians or government agencies with larger samples and perhaps expand on other social issues for which misinformation is a concern. Such work would extend the generalizability of our findings and address theoretical concerns of how partisanship and goal inferences work together with other factors to affect what people believe.

### Practical Implications

We also find merit in our results in terms of their implications for theory-based interventions and health practitioners. To our knowledge, we are the first to demonstrate that goal understanding processes matter for misinformation about COVID-19 among Republicans (and those not affiliated with a mainstream party). Those responsible for messages aimed at increasing belief in valid information about COVID-19 should recognize the need to address individuals’ pre-existing political views in order to prevent them from interpreting public health information as a political issue.

Exposure to attitudinally incongruent political information can elicit a type of biased information processing known as *motivated skepticism* [41]. If COVID-19 health information is viewed as a political issue, social media public health campaigns have the capacity to reinforce a pre-existing belief in misinformation rather than educating the public. Thus, future social media campaigns aimed at reducing the endorsement of misinformation should take into account the sentiments of their target audience’s inferences regarding their local government’s goals.

### Conclusions

A deeper understanding of the relationship among partisanship, goal understanding, and other cognitive processes would prove fruitful for our knowledge regarding how people process and endorse health misinformation. Such work would facilitate the development of effective social media public health interventions during the COVID-19 infodemic, and it would also uncover the mechanisms of goal understanding in message processing beyond interpersonal dyadic contexts.

## Appendix

Multimedia Appendix 1 Survey items.

## Abbreviations

H1: hypothesis 1
H2: hypothesis 2
LIWC: Linguistic Inquiry and Word Count
