# Supplementary material for: Constituents’ Inferences of Local Governments’ Goals and the Relationship Between Political Party and Belief in COVID-19 Misinformation: Cross-sectional Survey of Twitter Followers of State Public Health Departments
Source: JMIR Infodemiology. 2022 Feb 10;2(1):e29246. doi: 10.2196/29246 (PMC10014089; doi:10.2196/29246)
Supplement: Multimedia Appendix 1 [file infodemiology_v2i1e29246_app1.docx]

## **Appendix**

### **Measures**

#### ***Goal Inference Valence***

When your local government responded to the COVID-19 crisis, they did so with a few objectives (e.g., goals, purposes, end-states) in mind. They tried to achieve these objectives or goals via local policies.

We are interested in what goals you believe your local government are trying to achieve via their response to the COVID-19 crisis. That is, what were they trying to accomplish?

#### ***Endorsement in COVID-19 Misinformation*** (Parenthetical false versus true is for coding purposes only for our composite measure.)

Over the past few weeks, there has been conflicting information in media reports regarding COVID-19, also known as the novel coronavirus pandemic. The coronavirus has spread from to the United States prompting public officials to instill various social distancing regulations.

To the best of your knowledge, which of the following statements are true regarding COVID-19 transmissions?

| Children cannot get COVID-19 (false) |
| --- |
| Wearing a mask in public spaces protects against COVID-19 (true) |
| Cats and Dogs spread COVID-19 to humans (false) |
| A vaccine to cure COVID-19 is available (false at the time) |
| Thermal scanners can diagnose COVID-19 (false) |
| You can become infected from coming into close contact with others (true) |
| You can become infected by touching a surface the virus is on (true) |
| Hand washing with soap and water kills the disease (true) |
| Hand sanitizer kills the disease (true) |
| Wearing a face covering in public can cause carbon dioxide poisoning (false) |
